# Comparison of the perioperative complications of high intensity focused ultrasound vs. laparoscopic surgery for uterine fibroids: a retrospective study

**DOI:** 10.3389/fsurg.2025.1568000

**Published:** 2025-07-17

**Authors:** Li Hu, Chunling Fang, Nenghuan Tang, Fan Xu

**Affiliations:** ^1^ Department of Obstetrics and Gynecology, the Affiliated Nanchong Central Hospital of North Sichuan Medical College, Nanchong, Sichuan, China; ^2^ Department of Obstetrics and Gynecology, Affiliated Hospital of North Sichuan Medical College, Nanchong, Sichuan, China; ^3^ Department of Gynecology, Shanghai First Maternal and Infant Health Care Hospital, Shanghai, China

**Keywords:** high intensity focused ultrasound, laparoscopic surgery, perioperative complications, Clavien–Dindo classification, numerical rating scale (NRS)

## Abstract

**Objective:**

To compare the perioperative complications following high intensity focused ultrasound (HIFU) or laparoscopic surgery for uterine fibroids.

**Methods:**

A retrospective cohort was conducted involving patients with uterine fibroids (UFs) who underwent HIFU or laparoscopic surgery. The primary outcome was the incidence of perioperative complications. Secondary outcomes included the Numerical Rating Scale (NRS) for pain assessment, duration of hospital stay, hospitalization costs, and the incidence of short-term postoperative complications within 1 month. Univariate and multivariate logistic regression analyses were conducted to identify the influencing factors.

**Results:**

A total of 140 patients were included in the study, with an overall perioperative complication rate of 72.9%. Among them, 46 patients underwent HIFU treatment, while 94 underwent laparoscopic surgery. The HIFU group experienced significantly fewer total complications (52.2% vs. 83.0%, *P* < 0.001) and grade ≥ II complications (4.3% vs. 26.6%, *P* = 0.021) compared to the laparoscopic group. Further multivariate logistic regression revealed that treatment modality (Laparoscopic Surgery vs. HIFU: OR 5.48, 95% CI: 1.17–25.65, *P* = 0.031) was independent risk factors for grade ≥ II complications. Moreover, the HIFU group also experienced less pain on postoperative day 1, 2, and 3 compared to the laparoscopic surgery group (2.50 vs. 4.00, *P* < 0.001; 1.00 vs. 4.00, *P* < 0.001; 1.00 vs. 3.00, *P* < 0.001; respectively). Additionally, subgroup analyses showed that laparoscopic myomectomy (LM) group had fewer grade ≥ II complications than laparoscopic hysterectomy (LH) group (15.4% vs. 40.5%, *P* = 0.006).

**Conclusion:**

HIFU treatment is associated with a lower rate of perioperative complications, including grade ≥ II complications, reduced postoperative pain, shorter hospital stays, and lower hospitalization costs compared to laparoscopic surgery. These findings suggest that HIFU may serve as a viable alternative strategy for the management of uterine fibroids.

## 1 Introduction

Uterine fibroids (UFs) are the most common tumors in the female reproductive system (1). These tumors are characterized by symptoms such as heavy menstrual bleeding, pelvic pain, and fertility problems, all of which can significantly compromise the quality of life in affected individuals (2). Current management strategies for UFs primarily involve surgical interventions (3). With advancements in medical technology, minimally invasive and non-invasive treatment options, including laparoscopic hysterectomy (LH), laparoscopic myomectomy (LM), high intensity focused ultrasound (HIFU) have gained increasing popularity among patients (4).

Compared with traditional laparotomy, laparoscopic surgery offers several benefits, including shorter hospital stays, faster return to normal activities, reduced postoperative pain, and fewer perioperative complications (5). Common laparoscopic procedures for UFs include LH and LM, both of which have been extensively studied for their safety and efficacy (6). However, the choice of different treatments may lead to different complications. Some studies have reported that the main complications of LM include blood loss, fever, pain and adhesions (7), others have noted that the primary complications of LH are bleeding, direct injury, postoperative pain, and fever (8).

In recent years, HIFU has been increasingly utilized in the treatment of UFs. As a non-invasive modality, HIFU offers several advantages over traditional laparoscopic surgery, including reduced tissue trauma, faster recovery, and improved patient comfort (9–12). It serves as a promising alternative to conventional surgical procedures in appropriately selected patients (13). However, HIFU is also with some clinical complications, such as vaginal discharge, pain, nerve damage, and infection (14).

The comparison of complications between HIFU and laparoscopic surgery for UFs remains controversial. Liu et al. have concluded that HIFU has comparable adverse event rates to those of surgery (15). However, Wang et al. have shown that HIFU is associated with fewer clinical complications and adverse events compared to laparoscopic myomectomy (16). Therefore, we conducted a retrospective study to compare the incidence of perioperative complications, grade ≥ II complications, postoperative pain, hospital stays, and hospitalization costs between the HIFU treatment and laparoscopic surgery. Additionally, we performed a subgroup analysis to compare outcomes between LM and LH.

## 2 Material and methods

### 2.1 Study design and participants

This single-center retrospective study conducted at a tertiary care center from May 1, 2023 to January 1, 2024. This study was approved by the Institutional Review Board of Nanchong Central Hospital, and written informed consent was obtained from all participants. The primary outcome was the incidence of perioperative complications. Secondary outcomes included the Numerical Rating Scale (NRS) for pain assessment, duration of hospital stay, hospitalization costs, and the incidence of short-term postoperative complications within 1 month. Additionally, we performed a subgroup analysis to compare outcomes between LM and LH.

Inclusion and exclusion criteria for HIFU group: (1) uterine fibroids with clinical symptom, confirmed by imaging examination; (2) complete medical, imaging history and follow-up data; (3) able to lie prone for at least 1 h and stay awake during treatment; (4) safe acoustic access path to the lesion; (5) without a history of gynecological malignant tumors or other diseases and (6) without pregnancy. Inclusion and exclusion criteria for laparoscopic surgery group: (1) uterine fibroids with clinical symptom, confirmed by imaging examination; (2) complete medical, imaging history and follow-up data; (3) without a history of gynecological malignant tumors or other diseases; (4) without pregnancy and (5) without severe pelvic adhesions.

#### 2.1.1 Study procedures

HIFU group: Prior to the procedure, patients in the HIFU group completed bowel preparation. They were positioned supine on the treatment table, and intravenous access was established. Fibroids were localized through pre-treatment scanning, after which sedation and analgesia were provided if required. Contrast-enhanced ultrasound was employed to visualize blood flow signals within the target area, thereby delineating the treatment range. Parameter adjustments were made in accordance with the specific characteristics of the lesion. The procedure was concluded upon the detection of a significant alteration in grayscale within the target region (17).

#### 2.1.2 Laparoscopic surgery group

##### 2.1.2.1 LM group

Preoperative preparations included bowel preparation. Under general anesthesia, patients were positioned in the lithotomy position, and pneumoperitoneum was established. Oxytocin was administered intrauterinely, and fibroids were meticulously dissected and excised for pathological examination. The fibroid cavity and seromuscular layer were intermittently sutured with absorbable sutures. A standardized laparoscopic suturing technique utilizing 2-0 Vicryl sutures was employed consistently throughout the study. The drainage tube was removed on the second postoperative day. Prophylactic antibiotics were administered to prevent postoperative infections (18).

##### 2.1.2.2 LH group

Preoperative preparations included bowel preparation. During the procedure, patients were placed in the lithotomy position under general anesthesia, and a pneumoperitoneum was established. Initially, mobilize the bladder. Subsequently, coagulate and transect the uterine vessels. Proceed to separate the uterus and cervix from the vaginal apex. Finally, extract the uterus via the vaginal route and suture the vaginal cuff. The drainage tube was removed on the second postoperative day, and prophylactic antibiotics were administered to prevent postoperative infections (19).

### 2.2 Collecting and processing data

Perioperative complications were collected via reviewed medical records until the time of discharge. All complications were graded using the Clavien–Dindo classification system and independently assessed by a clinician with over 10 years of clinical experience. The Clavien–Dindo classification categorizes complications into five grades (20): Grade I: complications requiring only antiemetics, antipyretics, analgesics, diuretics, electrolytes, or physiotherapy. Grade II: complications requiring pharmacological treatment beyond those permitted for grade I, including blood transfusions or total parenteral nutrition. Grade IIIa: complications requiring surgical, endoscopic, or radiologic intervention without general anesthesia. Grade IIIb: intervention under general anesthesia. Grade IVa: life-threatening complications requiring intensive care unit (ICU) management with single-organ dysfunction. Grade IVb:life-threatening complications with multi-organ dysfunction. Grade V: death. Furthermore, we dichotomized the Clavien–Dindo grade into Clavien–Dindo grade < II and Clavien–Dindo grade ≥ II to potential factors associated with more severe perioperative complications.

Baseline characteristics and surgery-related data were also collected via reviewed medical records, which including age, body mass index (BMI), marital status, smoking history, alcohol consumption, presence of complication, location of largest fibroid (i.e., anterior wall, posterior wall or other), number of fibroids (single or multiple), largest fibroid volume (length × width × height × 0.523), uterine volume (length × width × thickness × 0.523), history of previous abdominal surgery (yes or no), hospitalization costs and hospital stays.

The pain condition of patients was assessed by the Numerical Rating Scale (NRS) on first day before the surgery, the day of surgery, as well as postoperative day 1, 2, and 3. Furthermore, all patients were followed up at 1 month after the procedure to assessed the short-term postoperative complications within 1 month via telephone. Patients who failed to respond after three times attempts were defined as lost to follow-up.

### 2.3 Statistical analysis

SPSS 27.0 was applied for statistical analysis. Continuous variables were expressed as mean ± standard deviation (SD) for normally distributed data or median with interquartile range (IQR) for non-normally distributed data. Categorical variables were presented as frequencies and percentages. The Shapiro–Wilk test was used to assess the normality of continuous variables. For non-normally distributed continuous variables, the Mann–Whitney *U* test was used. The Fisher's exact test or Chi-square test was used for categorical variables. Besides, univariate and multivariate logistics regression analyses was utilized to identify influencing factors for more severe perioperative complications for UFs following HIFU or laparoscopic surgery treatments. All variables with *P*-value < 0.05 in univariate logistics analysis were eligible for inclusion as potential predictors in the multivariate logistic. A two-sided *P*-value of <0.05 indicated statistical significance.

## 3 Results

### 3.1 Base characteristics

A total of 140 patients were included in this study, with 46 (32.9%) in the HIFU group and 94 (67.1%) in the laparoscopic surgery group. All patients completed the 1-month postoperative follow-up. The baseline demographic and clinical characteristics of the patients are summarized in Table 1. There were no significant differences between the two groups (HIFU group vs. laparoscopic surgery group) in terms of in BMI, marital status, smoking history, alcohol consumption, location of largest fibroid and number of fibroids (all *P* > 0.05). However, significant differences were observed in age (43.00 vs. 46.00 years, *P* = 0.023), largest fibroid volume (55.15 vs.143.36 cm^3^, *P* = 0.004), and uterine volume (146.82 vs. 256.56 cm^3^, *P* < 0.001) between HIFU group and laparoscopic surgery group. Furthermore, we conducted the subgroup analysis for laparoscopic surgery group, of those, 42 patients underwent LH and 52 patients underwent LM. The detail baseline demographic as shown in Supplementary Appendix 1.

**Table 1:** Baseline characteristics of the patients between the HIFU and Surgery group.

Characteristic	HIFU (*N* = 46)	Surgery (*N* = 94)	*P*
Demographic characteristics			
Age (years)	43.00 (38.75–46.00)	46.00 (40.75–49.00)	0.023
BMI (kg/m^2^)	24.80 (21.90–27.13)	25.00 (22.68–27.43)	0.274
Marital status *n* (%)			0.737
Married	44.00 (95.7)	87.00 (92.6)	
Unmarried	2.00 (4.3)	7.00 (7.4)	
Smoking *n* (%)			0.639
Yes	3.00 (6.5)	3.00 (3.2)	
No	43.00 (93.5)	91.00 (96.8)	
Alcohol consumption *n* (%)			0.639
Yes	3.00 (6.5)	3.00 (3.2)	
No	43.00 (93.5)	91.00 (96.8)	
Fibroid assessment			
Location of largest fibroid *n* (%)			0.915
Anterior wall	17.00 (34.1)	30.00 (34.1)	
Posterior wall	16.00 (35.6)	33.00 (37.5)	
Other	12.00 (26.7)	25.00 (28.4)	
No. of fibroids *n* (%)			<0.127
Single	11.00 (23.9)	23.00 (24.3)	
Multiple	35.00 (76.1)	66.00 (70.2)	
Largest fibroid volume			0.004
Mean (cm^3^)	55.15 (21.93–68.62)	143.36 (30.71–221.77)	
Uterine volume			<0.001
Mean (cm^3^)	146.82 (108.93–280.09)	256.56 (144.49–398.95)	
Surgery-related			
History of previous abdominal surgery *n* (%)			0.229
No	27.00 (58.7)	45.00 (47.9)	
Yes	19.00 (41.3)	49.00 (52.1)	
Hospitalization cost	14,500.09 (13,954.10–15,807.20)	17,354.86 (15,482.99–18,994.26)	<0.001
Hospital stays	4.00 (4.00–5.00)	7.00 (7.00–8.25)	<0.001

Data are Median (interquartile range) values, except as noted. Abbreviations: HIFU, high-intensity focused ultrasound; C–D grade, Clavien–Dindo grade; BMI, body mass index.

### 3.2 Complications of different treatment

**Figure 1:**
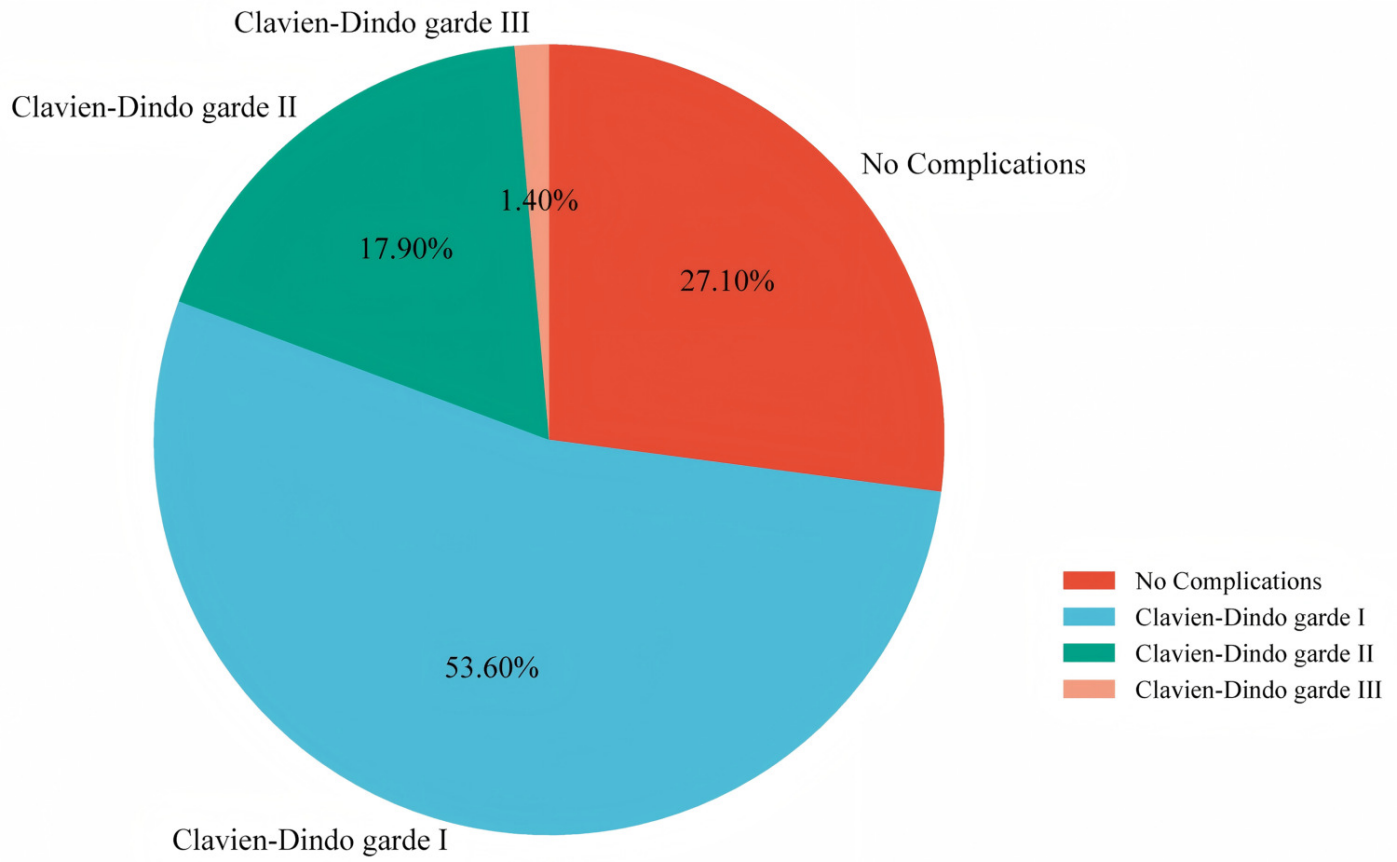
The complication rate of 140 patients with uterine fibroids.

In this study, the overall perioperative complications rate in the all treatment strategies for UFs was 72.9%, with Clavien–Dindo grade I complications accounting for 75 (53.6%), grade II for 25 (17.9%), and grade III for 2 (1.4%) (Figure 1). No grade IV or V complications occurred in our study. Among the two grade III complications observed, one patient required local anesthesia for drain fixation due to leakage, while another underwent interventional therapy to address postoperative lower limb venous thrombosis. Compared with laparoscopic surgery, HIFU was associated with a significantly lower incidence of overall perioperative complications (52.2% vs. 83.0%, *P* < 0.001) and fewer grade ≥ II complications (4.3% vs. 26.6%, *P* = 0.021). Compared to the grade < II group, the grade ≥ II group had larger maximum fibroid volume (*P* = 0.012) and greater uterine volume (*P* < 0.001) (Table 2). Based on the Clavien–Dindo classification, in the HIFU group, there were fewer grade ≥ II complications (4.3% vs. 26.6%) and more grade < II complications (95.7% vs. 73.4%) than in the laparoscopic surgery group, with statistically significant differences (*P* < 0.021) (Supplementary Appendix 2).

**Table 2:** Baseline characteristics of the patients between the C-D grade <II and C-D grade ≥II group.

Characteristic	C–D grade < II (*N* = 113)	C-Dgrade ≥ II (*N* = 27)	*P*
Demographic characteristics			
Age (years)	44.00 (39.00–48.00)	46.00 (43.00–49.00)	0.398
BMI (kg/m^2^)	24.90 (22.15–27.25)	26.00 (23.20–27.50)	0.494
Marital status *n* (%)			1.000
Married	105.00 (97.2)	26.00 (96.3)	
Unmarried	3.00 (2.8)	1.00 (3.7)	
Smoking *n* (%)			0.487
Yes	107.00 (94.79)	27.00 (100.0)	
No	6.00 (5.3)	0.00 (0.0)	
Alcohol consumption *n* (%)			0.487
Yes	107.00 (94.79)	27.00 (100.0)	
No	6.00 (5.3)	0.00 (0.0)	
Fibroid assessment			
Location of largest fibroid *n* (%)			0.401
Anterior wall	39.00 (36.4)	8.00 (30.8)	
Posterior wall	41.00 (38.3)	8.00 (30.8)	
Other	27.00 (25.2)	10.00 (38.5)	
No. of fibroids *n* (%)			0.410
Single	30.00 (26.5)	4.00 (14.8)	
Multiple	79.00 (69.9)	22.00 (81.5)	
Largest fibroid volume			0.012
Mean (cm^3^)	94.51 (22.29–138.97)	198.70 (54.11–253.66)	
Uterine volume			<0.001
Mean (cm^3^)	184.07 (113.92–315.00)	360.70 (224.96–571.83)	
Surgery-related			
History of previous abdominal surgery *n* (%)			0.249
No	60.00 (53.1)	11.00 (40.7)	
Yes	53.00 (46.9)	16.00 (59.3)	
Hospitalization cost	15,934.14 (14,210.53–17,908.86)	18,676.91 (16,272.15–23,671.50)	0.090
Hospital stays	7.00 (5.00–8.00)	8.00 (7.00–9.00)	0.226

Data are Median (interquartile range) values, except as noted. Abbreviations: HIFU, high-intensity focused ultrasound; C–D grade, Clavien–Dindo grade; BMI, body mass index.

**Figure 2:**
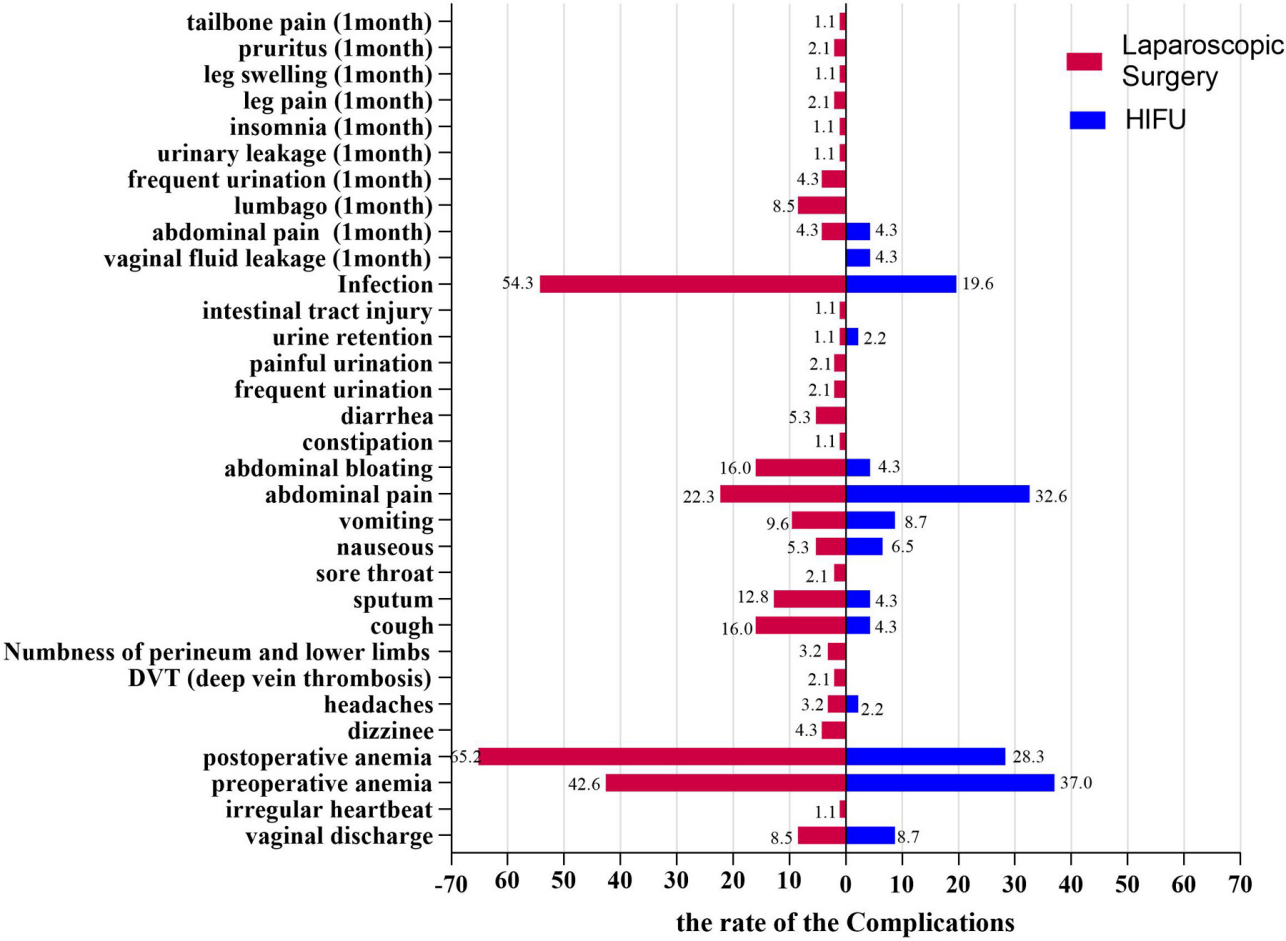
Comparison of two different treatment modalities for preoperative complications and short term postoperative complications (one month after treatment) in patients with fibroids.

The most common perioperative complications in the HIFU group included preoperative anemia (37.00%), abdominal pain (32.6%), postoperative anemia (28.3%), infection (19.6%) and vomiting (8.7%). In the laparoscopic surgery group, the most frequent complications were postoperative anemia (65.2%), infection (54.3%), preoperative anemia (42.6%), abdominal pain (22.3%), cough (16.0%), and abdominal bloating (16.0%) (Supplementary Appendix 2, Figure 2). Furthermore, the overall rate of postoperative complications within 1 month after the procedure was 14.3%. In the HIFU group, the most commonly reported complications were vaginal fluid leakage (4.3%) and abdominal pain (4.3%). In contrast, the laparoscopic surgery group experienced lumbago (8.5%), abdominal pain (4.3%), frequent urination (4.3%), leg pain (2.1%) and pruritus (2.1%) (Supplementary Appendix 2, Figure 2). Moreover, subgroup analysis revealed that patients in the LH group had a significantly higher incidence of Clavien–Dindo grade ≥ II complications compared with those who underwent LM (40.5% vs. 15.4%, *P* = 0.006). Detailed complication profiles are presented in Supplementary Appendix 3.

### 3.3 Risk factors for Clavien–Dindo grade ≥ II complications

In the univariate logistic regression analysis, HIFU treatment was significantly associated with a reduced risk of Clavien–Dindo grade ≥ II perioperative complications compared to laparoscopic surgery (OR: 7.97; 95% CI: 1.80–35.34; *P* = 0.006). In addition, both maximum fibroid diameter (OR: 1.25; 95% CI: 1.05–1.49; *P* = 0.011) and maximum uterine diameter (OR: 1.31; 95% CI: 1.06–1.61; *P* = 0.001) were significantly associated with an increased risk of Clavien–Dindo grade ≥ II perioperative complications. Specifically, each 1 cm increase in maximum uterine diameter was associated with a 31% higher risk, and each 1 cm increase in maximum fibroid diameter was associated with a 25% higher risk.

In the multivariate logistic regression analysis, HIFU treatment also showed similar relationship with Clavien–Dindo grade ≥ II perioperative complications (OR: 5.48; 95% CI: 1.17–25.65; *P* = 0.031). However, the associations between the risk of Clavien–Dindo grade ≥ II perioperative complications and the maximum diameter of the uterus (OR: 1.17; 95% CI: 0.93–1.47; *P* = 0.176) and fibroid (OR: 1.13; 95% CI: 0.91–1.39; *P* = 0.266) present no statistically significant (Table 3).

**Table 3:** Univariate and multivariate logistics regression analysis of risk factors associated with complications Clavien-Dindo grade ≥II.

Variables	Univariate logistics regression			Multivariate logistics regression		
	OR	95% CI	*P*	OR	95% CI	*P*
Treatment modality (surgery vs. HIFU)	7.97	1.80–35.34	0.006	5.48	1.17–25.65	0.031
Age (years)	1.07	0.98–1.15	0.122	NA	NA	NA
BMI (kg/m^2^)	1.04	0.93–1.16	0.512	NA	NA	NA
Marital status (unmarried vs. married)	0.51	0.60–4.22	0.528	NA	NA	NA
Maximum diameter of the uterus	1.31	1.06–1.61	0.001	1.17	0.93–1.47	0.176
Maximum diameter of uterine fibrods	1.25	1.05–1.49	0.011	1.13	0.91–1.39	0.266
Number of leiomyomas (multiple vs. single)	2.09	0.67–6.57	0.208	NA	NA	NA
Location of the main fibroids (*n*, %)						
Anterior wall	ref	ref	ref	NA	NA	NA
Posterior wall	0.95	0.33–2.78	0.927	NA	NA	NA
Other	1.81	0.63–5.17	0.271	NA	NA	NA

Abbreviations: HIFU, high-intensity focused ultrasound; BMI, body mass index; NA, not applicable; OR, odd rations; 95% CI, 95% confidence interval.

### 3.4 The pain and surgery-related

In this study, we compared pain levels of patients between two groups, which indicated that the HIFU group experienced significantly lower pain scores than laparoscopic surgery group on postoperative days 1, 2, and 3 (2.50 vs. 4.00, *P* < 0.001; 1.00 vs. 4.00, *P* < 0.001; 1.00 vs. 3.00, *P* < 0.001, respectively) (Table 4). Further analysis showed that the HIFU group had lower hospitalization costs (¥14,500.09 vs. ¥17,354.86, *P* < 0.001) ad shorter hospital stays (4.00 vs. 7.00 days, *P* < 0.001) compared to the laparoscopic surgery group (Table 1). Subgroup analysis revealed that the LM group exhibited lower hospitalization costs (¥16,487.72 vs. ¥18,038.64, *P* = 0.017) than the LH group (Supplementary Appendix 1).

**Table 4:** The pain evaluated by numerical rating scale (NRS) score between the HIFU and Surgery group.

Variables	HIFU (*N* = 46)	Surgery (*N* = 94)	*P*
Baseline (preoperative pain)	1.50 (0.00–3.00)	1.00 (0.00–3.00)	0.877
Pain on the day of surgery	5.00 (3.00–7.00)	4.00 (3.00–7.00)	0.795
Pain on the first day of surgery	2.50 (1.00–4.00)	4.00 (3.00–6.00)	<0.001
Pain on the second day of surgery	1.00 (0.00–2.00)	4.00 (2.50–5.00)	<0.001
Pain on the third day of surgery	1.00 (0.00–2.25)	3.00 (1.75–4.00)	<0.001

The data were present as median (interquartile range). Abbreviations: NRS, numerical rating scale score; HIFU, high-intensity focused ultrasound.

## 4 Discussion

In this study, we aimed to investigate the perioperative complications between the HIFU and surgery groups. The results showed that HIFU treatment may have less perioperative complication rate, Clavien–Dindo grade ≥ II perioperative complications, pain level, hospital stays, and hospitalization costs than laparoscopic surgery. Further logistic regression analyses indicated that treatment modalities were linked to the risk of Clavien–Dindo grade ≥ II perioperative complications.

HIFU and laparoscopic surgery are the two most common treatments for UFs in China. Recently, some studies have shown the safety and efficacy of them (21, 22). However, there is still a lack of literature comparing the incidence of complications associated with these two treatments. Previous studies have reported complication rates of 0.4%–28.1% for HIFU and 11.6%–56.2% for laparoscopic surgery, suggesting that HIFU may be associated with a lower risk of postoperative complications (15, 23). Consistent with previous studies, our findings also demonstrated a lower incidence of complications in the HIFU group. Besides, long-term outcomes also favor HIFU, with literature showing low fibroid recurrence and improved quality of life (16, 24). These results provide valuable evidence to support clinical decision-making when developing individualized treatment plans and offer patients with UFs a less invasive, lower-risk therapeutic option.

HIFU serves as a minimally invasive therapeutic modality for uterine fibroids (UFs), utilizing ultrasonic energy to precisely target fibroids and induce coagulative necrosis (25). The complications associated with HIFU predominantly arise from its thermal and mechanical effects (26). Common adverse effects observed in patients undergoing HIFU treatment include anemia, abdominal pain, infection, and vomiting. The mechanism underlying HIFU involves the induction of pressure changes in tissues by ultrasound, which converts mechanical energy into heat through friction, thereby achieving coagulative necrosis of the target tissue while sparing the surrounding tissues (27). Nevertheless, the mechanical effects of HIFU can result in complications such as skin burns, infections, vascular disruption, and vessel occlusion. The severity of these mechanical effects is contingent upon treatment parameters, including ultrasonic frequency, intensity, and duration, as well as the characteristics of the fibroids, such as size and blood flow (28). In our study, no instances of skin burns were observed, likely due to the meticulous control of treatment parameters, which mitigated the risk of such complications. HIFU treatments are usually carried out with the patient lightly sedated (29), and postoperative vomiting may result from narcotics affecting gastrointestinal function.

Perioperative complications in laparoscopic surgery patients mainly stem from mechanical injury and infection risks, which may be related to surgical instruments, the operative environment, or endogenous flora (30). The most common complications in the Laparoscopic surgical group anemia, infection, abdominal pain, cough and abdominal bloating. Laparoscopic surgery is more invasive than HIFU. Intraoperative blood loss can reduce hemoglobin levels, potentially leading to anemia. Laparoscopic surgery is invasive and demands high sterilization standards for surgical instruments and sites. During surgery, any breach of aseptic technique may result in infection. Abdominal pain frequently arises from the surgical incision, in addition to tissue and organ damage associated with trocar insertion and the manipulation of instruments. As laparoscopic surgeries are performed under general, anesthesia postoperative coughing and sputum production are usually due to its impact on respiratory function (31). Abdominal bloating may due to gas accumulation in the gastrointestinal tract from pneumoperitoneum and instrument manipulation (32).

Furthermore, we found that treatment modality, the maximum diameter of the uterus or fibroid are linked to more severe complications. In laparoscopic surgery, large fibroids can hinder instrument movement, complicate tissue removal, and increase procedure time, bleeding, and complication risks. In HIFU therapy, the maximum diameter of the uterus or fibroid can reduce treatment effectiveness due to greater ultrasound penetration distance and require longer treatment, raising the risk of skin burns and pain. However, these associations did not persist after adjusting for confounders, indicating that other factors such as surgical technique or patient physiology may modulate their effects (33). But, the association between treatment modalities and more severe complications were also present in multivariate analysis adjusting the maximum diameter of the uterus or fibroid, which also indicated that HIFU has less complications than laparoscopic surgery among more severe complications.

High Intensity Focused Ultrasound reduces postoperative pain may due to its non-invasive nature. Liu et al. also reported lower pain scores in patients treated with HIFU compared to those who underwent surgical procedures (15). This reduction in pain can be attributed to HIFU's ability to avoid abdominal incisions and precisely target fibroids, thereby minimizing trauma to surrounding nerves and tissues (10). Pain levels typically peak on the day of the procedure and the first postoperative day, subsequently declining to acceptable levels. Pain scores are generally classified as follows: 0 indicates no pain, 1–3 indicates mild pain, 4–6 indicates moderate pain, and 7–10 indicates severe pain (34). Wu et al. also reported that a Numeric Rating Scale (NRS) score of ≥4 represents the threshold for moderate to severe pain (35). The incidence of short-term complications within 1 month did not differ significantly between the groups, likely due to the limited duration of follow-up.

Subgroup analyses revealed comparable perioperative complication rates between LH and LM (*P* = 0.934), but the LH group exhibited a higher rate of grade ≥ II complications (*P* = 0.006). Tsai et al. reported similar findings, with no significant difference in perioperative morbidity between the two groups (36). The complication level of LH was less than that of LM, possibly because of the extended operation duration and significant wound size of LH. Some studies have reported that LH may cause more trauma (16), increasing the risk of damage to neighboring organs such as the intestines, which was observed in one case in our study. One month post-procedure, the incidence of low back pain was significantly lower in the LM group compared to LH (*P* = 0.030), possibly due to partial suspension of the vaginal cuff in hysterectomy patients to prevent prolapse.

This study has several limitations. First, its retrospective, single-center design with a small sample size may limit generalizability. Future prospective multicenter studies are needed to thoroughly explore the complications of both techniques. However, our study offers initial evidence that HIFU may reduce complications. Second, the focus on short-term outcomes and lack of long-term complication data limit assessment of durability and late effects. The short-term complications suggest HIFU aids quick recovery, but long-term follow-up is needed to overall assess its safety compared to surgery. Third, although the Numeric Rating Scale (NRS) is widely used for pain assessment, its reliance on cognitive ability may bias results, especially in patients with limited cognitive function. Fourth, the study population skewed younger, which may influence complication rates and treatment selection. Finally, telephone follow-up may introduce recall bias.

In conclusion, HIFU is associated with fewer complications and lower Clavien–Dindo grades compared to laparoscopic surgery, as well as less postoperative pain and shorter hospital stays. Subgroup analyses show LH and LM have comparable complication incidences, but LH is associated with more severe complications. These findings provide valuable insights for clinicians and surgical teams in selecting appropriate uterine fibroid treatments and highlight HIFU as an effective, minimally invasive alternative.

## Data Availability

The data analyzed in this study is subject to the following licenses/restrictions: If you need information about the original data, please contact the first author. Requests to access these datasets should be directed to 2321902532@qq.com.
