# Supplementary material for: Comparison of the perioperative complications of high intensity focused ultrasound vs. laparoscopic surgery for uterine fibroids: a retrospective study
Source: Front Surg. 2025 Jul 17;12:1568000. doi: 10.3389/fsurg.2025.1568000 (PMC12310626; doi:10.3389/fsurg.2025.1568000)
Supplement: Supplementary file 1 [file Datasheet1.zip › Revised supplementary tables/Appendix 1 final version.doc]

Appendix 1 Baseline characteristics of the patients between the Hysterectomy and Myomectomy group.

| **Characteristic** | **Hysterectomy(N=42)** | **Myomectomy(N=52）** | ***P*** |
| --- | --- | --- | --- |
| **Demographic characteristics** |  |  |  |
| Age — year | 48.00(44.75-50.00) | 43.00(38.00-47.75) | ＜0.001 |
| BMI(kg/m2) | 24.50(23.08-27.43) | 24.45(22.53-27.75) | 0.290 |
| Marital status n(%) |  |  | 1.000 |
| Married | 39.00(92.9) | 48.00(92.3) |  |
| Unmarried | 3.00(7.1) | 4.00(7.7) |  |
| Smoking n(%) |  |  | 1.000 |
| Yes | 41.00(97.6) | 50.00(96.2) |  |
| No | 1.00(2.4) | 2.00(3.8) |  |
| Alcohol consumption n(%) |  |  | 0.321 |
| Yes | 42.00(100.00) | 49.00(94.2) |  |
| No | 0.00(0.0) | 3.00(5.8) |  |
| **Fibroid assessment** |  |  |  |
| Location of largest fibroid n(%) |  |  | 0.258 |
| Anterior wall | 11.00(26.2) | 19.00(36.5) |  |
| Posterior wall | 18.00(42.9) | 15.00(28.8) |  |
| Other | 9.00(21.4) | 16.00(30.8) |  |
| No. of fibroids n(%) |  |  | 0.869 |
| Single | 9.00(21.4) | 14.00(25.9) |  |
| Multiple | 30.00(71.4) | 36.00(66.7) |  |
| Largest fibroid volume |  |  | 0.356 |
| Mean — cm3 | 104.12(33.38-164.95) | 165.66(30.37-258.03) |  |
| Uterine volume |  |  | 0.132 |
| Mean — cm3 | 290.56(194.18-389.00) | 207.50(117.18-427.21) |  |
| **Surgery-related** |  |  |  |
| History of previous abdominal surgery n(%) |  |  | 0.106 |
| No | 24.00(57.1) | 21.00(40.4) |  |
| Yes | 18.00(42.9) | 31.00(59.6) |  |
| Hospitalization cost | 18038.64(16345.86-19618.17) | 16487.72(14871.19-18463.84) | 0.017 |
| Hospital stay | 7.00(7.00-8.25) | 8.00(7.00-8.75) | 0.689 |

Abbreviations: BMI: body mass index; C-D grade:Clavien - Dindo grade. Data are Median (interquartile range) values,except as noted.
