# Supplementary material for: Comparison of the perioperative complications of high intensity focused ultrasound vs. laparoscopic surgery for uterine fibroids: a retrospective study
Source: Front Surg. 2025 Jul 17;12:1568000. doi: 10.3389/fsurg.2025.1568000 (PMC12310626; doi:10.3389/fsurg.2025.1568000)
Supplement: Supplementary file 1 [file Datasheet1.zip › Revised supplementary tables/Appendix 2 final version.doc]

Appendix 2 Perioperative complications and postoperative complications between the HIFU and Surgery group.

| **Complication(%)** | **HIFU(N=46)** | **Surgery(N=94)** |
| --- | --- | --- |
| **Cardiovascular system** |  |  |
| Vaginal discharge | 4.00(8.7%) | 8.00(8.5%) |
| Irregular heartbeat | 0.00(0.0%) | 1.00(1.1%) |
| Preoperative anemia | 17.00(37.0%) | 40.00(42.6%) |
| Postoperative anemia | 13.00(28.3%) | 60.00(65.2%) |
| Dizziness | 0.00(0.0%) | 4.00(4.3%) |
| Headache | 1.00(2.2%) | 3.00(3.2%) |
| DVT | 0.00(0.0%) | 2.00(2.1%) |
| **Motion system** |  |  |
| Numbness of perineum and lower limbs (paresthesia) | 0.00(0.0%) | 3.00(3.20%) |
| Respiratory system |  |  |
| Cough | 2.00(4.3%) | 15.00(16.0%) |
| Sputum | 2.00(4.3%) | 12.00(12.8%) |
| Sore throat | 0.00(0.0%) | 2.00(2.1%) |
| **Digestive system** |  |  |
| Nauseous | 3.00(6.5%) | 5.00(5.3%) |
| Vomiting | 4.00(8.7%) | 9.00(9.60%) |
| Abdominal pain | 15.00(32.6%) | 21.00(22.3%) |
| Abdominal bloating | 2.00(4.3%) | 15.00(16.0%) |
| Constipation | 0.00(0.0%) | 1.00(1.10%) |
| Diarrhea | 0.00(0.0%) | 5.00(5.3%) |
| **Urinary system** |  |  |
| Frequent urination | 0.00(0.0%) | 2.00(2.1%) |
| Painful urination | 0.00(0.0%) | 2.00(2.1%) |
| Urine retention | 1.00(2.2%) | 1.00(1.1%) |
| **Impairment** |  |  |
| Intestinal tract injury | 0.00(0.0%) | 1.00(1.1%) |
| **Other** |  |  |
| Infection | 9.00(19.6%) | 51.00(54.3%) |
| **Postoperative complications (one month)** |  |  |
| Vaginal fluid leakage | 2.00(4.3%) | 0.00(0.0%) |
| Abdominal pain | 2.00(4.3%) | 4.00(4.3%) |
| Lumbago | 0.00(0.0%) | 8.00(8.5%) |
| Frequent urination | 0.00(0.0%) | 4.00(4.3%) |
| Urinary leakage | 0.00(0.0%) | 1.00(1.1%) |
| Constipation | 0.00(0.0%) | 1.00(1.1%) |
| Leg pain | 0.00(0.0%) | 2.00(2.1%) |
| Leg swelling | 0.00(0.0%) | 1.00(1.1%) |
| Pruritus | 0.00(0.0%) | 2.00(2.1%) |
| Tailbone pain | 0.00(0.0%) | 1.00(1.1%) |
| C-D grade |  | P=0.021 |
| <II | 44.00(95.7%) | 69.00(73.4%) |
| ≥II | 2.00(4.3%) | 25.00(26.6%) |

Abbreviations: HIFU: high-intensity focused ultrasound; DVT: Deep venous thrombosis; C-D grade: Clavien - Dindo grade.
