# Supplementary material for: Comparison of the perioperative complications of high intensity focused ultrasound vs. laparoscopic surgery for uterine fibroids: a retrospective study
Source: Front Surg. 2025 Jul 17;12:1568000. doi: 10.3389/fsurg.2025.1568000 (PMC12310626; doi:10.3389/fsurg.2025.1568000)
Supplement: Supplementary file 1 [file Datasheet1.zip › Revised supplementary tables/Appendix 3 final version.doc]

Appendix 3 Perioperative complications and postoperative complications (one month) between the Hysterectomy and Myomectomy group.

| **Complicationn(%)** | **Hysterectomy(N=42)** | **Myomectomy(N=52）** |
| --- | --- | --- |
| **Cardiovascular system** |  |  |
| Vaginal discharge | 1.00(2.4) | 7.00(13.5) |
| Irregular heartbeat | 1.00(2.4) | 0.00(0.0) |
| Preoperative anemia | 22.00(52.4) | 17.00(32.7) |
| Postoperative anemia | 31.00(73.8) | 29.00(55.8) |
| Dizziness | 1.00(2.4) | 3.00(5.8) |
| Headache | 1.00(2.4) | 1.00(1.9) |
| DVT | 0.00(0.0) | 2.00(3.8) |
| **Motion system** |  |  |
| Numbness of perineum and lower limbs (paresthesia) | 2.00(4.8) | 1.00(1.9) |
| **Respiratory system** |  |  |
| Cough | 9.00(21.4) | 6.00(11.5) |
| Sputum | 8.00(19.0) | 4.00(7.7) |
| Sore throat | 1.00(2.4) | 1.00(1.9) |
| **Digestive system** |  |  |
| Nauseous | 1.00(2.4) | 4.00(7.7) |
| Vomiting | 4.00(9.5) | 5.00（9.6） |
| Abdominal pain | 8.00(19.0) | 13.00(25.0) |
| Abdominal bloating | 7.00(16.7) | 8.00(15.4) |
| Constipation | 1.00(2.4) | 0.00(0.0) |
| Diarrhea | 3.00(7.1) | 2.00(3.8) |
| **Urinary system** |  |  |
| Frequent urination | 2.00(4.8) | 0.00(0.0) |
| Painful urination | 2.00(4.8) | 0.00(0.0) |
| Urine retention | 1.00(2.4) | 0.00(0.0) |
| **Impairment** |  |  |
| Intestinal tract injury | 1.00(2.4) | 0.00(0.0) |
| **Other** |  |  |
| Infection | 23.00(52.9) | 27.00(51.9) |
| **Postoperative complications (one month)** |  |  |
| Abdominal pain | 3.00(7.1) | 1.00(1.9) |
| Lumbago | 7.00(16.7) | 1.00(1.9) |
| Frequent urination | 4.00(9.5) | 1.00(1.9) |
| Urinary leakage | 1.00(2.4) | 0.00(0.0) |
| Constipation | 1.00(2.4) | 0.00(0.0) |
| Leg pain | 2.00(4.8) | 0.00(0.0) |
| Leg swelling | 0.00(0.0) | 1.00(1.9) |
| Pruritus | 0.00(0.0) | 2.00(3.8) |
| C-D grade |  | P < 0.006 |
| <II | 25.00(59.5) | 44.00(84.6) |
| ≥II | 17.00(40.5) | 8.00(15.4) |

Abbreviations:HIFU: high-intensity focused ultrasound; DVT: Deep venous thrombosis; C-D grade: Clavien - Dindo grade.
